# A novel encoding element for robust pose estimation using planar fiducials

**DOI:** 10.3389/frobt.2022.838128

**Published:** 2022-08-24

**Authors:** David D. W. Rijlaarsdam, Martin Zwick, J.M. (Hans) Kuiper

**Affiliations:** ^1^ Faculty of Aerospace Engineering, Delft University of Technology, Delft, Netherlands; ^2^ European Space Research and Technology Centre (ESTEC), European Space Agency, Noordwijk, Netherlands

**Keywords:** Fiducial markers (FMs), pose estimation, encoding element, pose ambiguity elimination, monocular 3D motion estimation, robotic perception, navigation

## Abstract

Pose estimation in robotics is often achieved using images from known and purposefully applied markers or fiducials taken by a monocular camera. This low-cost system architecture can provide accurate and precise pose estimation measurements. However, to prevent the restriction of robotic movement and occlusions of features, the fiducial markers are often planar. While numerous planar fiducials exist, the performance of these markers suffers from pose ambiguities and loss of precision under frontal observations. These issues are most prevalent in systems with less-than-ideal specifications such as low-resolution detectors, low field of view optics, far-range measurements etc. To mitigate these issues, encoding markers have been proposed in literature. These markers encode an extra dimension of information in the signal between marker and sensor, thus increasing the robustness of the pose solution. In this work, we provide a survey of these encoding markers and show that existing solutions are complex, require optical elements and are not scalable. Therefore, we present a novel encoding element based on the compound eye of insects such as the Mantis. The encoding element encodes a virtual point in space in its signal without the use of optical elements. The features provided by the encoding element are mathematically equivalent to those of a protrusion. Where existing encoding fiducials require custom software, the projected virtual point can be used with standard pose solving algorithms. The encoding element is simple, can be produced using a consumer 3D printer and is fully scalable. The end-to-end implementation of the encoding element proposed in this work significantly increases the pose estimation performance of existing planar fiducials, enabling robust pose estimation for robotic systems.

## 1 Introduction

Pose estimation is an essential capability for many robotics systems (Zhou and Roumeliotis, 2008; Janabi-Sharifi and Marey, 2010; Romero-Ramire et al., 2019; Hietanen et al., 2021). For the purpose of this paper, pose estimation is defined as the six degree of freedom (DOF) transformation (i.e., position and orientation) between a sensor and a certain reference frame. To determine the 6 DOF pose in a robotic system, methods such as Simultaneous Localization and Mapping (SLAM) may be used. In scenarios where additional robustness is required or these methods are not feasible, a monocular camera is often used in combination with fiducial markers (Romero-Ramirez et al., 2018). Fiducial markers provide known feature correspondences that can be used as input to a Perspective-n-Point (PnP) solving algorithm. It can be shown that four co-planar and not co-linear points are sufficient to provide a unique solution to the PnP problem (Fischler and Bolles (1981)), thus most fiducial markers provide at least four distinct features.

In many applications fiducial markers are required to be planar. This can be due to volume constraints, the need for the prevention of occlusions or blocking of robotic movement, etc. Numerous planar markers exist for robotic systems (Kato and Billinghurst, 1999; Fiala, 2005; Olson, 2011; Garrido-Jurado et al., 2014). These markers are easy to produce, provide scalability and have low complexity. However, their performance suffers from pose ambiguities and loss of precision or jitter under frontal observations. While some attempts have been made to mitigate these issues in planar fiducials by providing better localisation of features, these markers still suffer from pose ambiguities (Benligiray et al., 2019). Both pose ambiguity and loss of precision under frontal observations affect the end-to-end performance of the pose estimation system and can cause failure of the pose solution. Both these issues are more severe under the influence of noise on the feature point locations.

**FIGURE 1:**
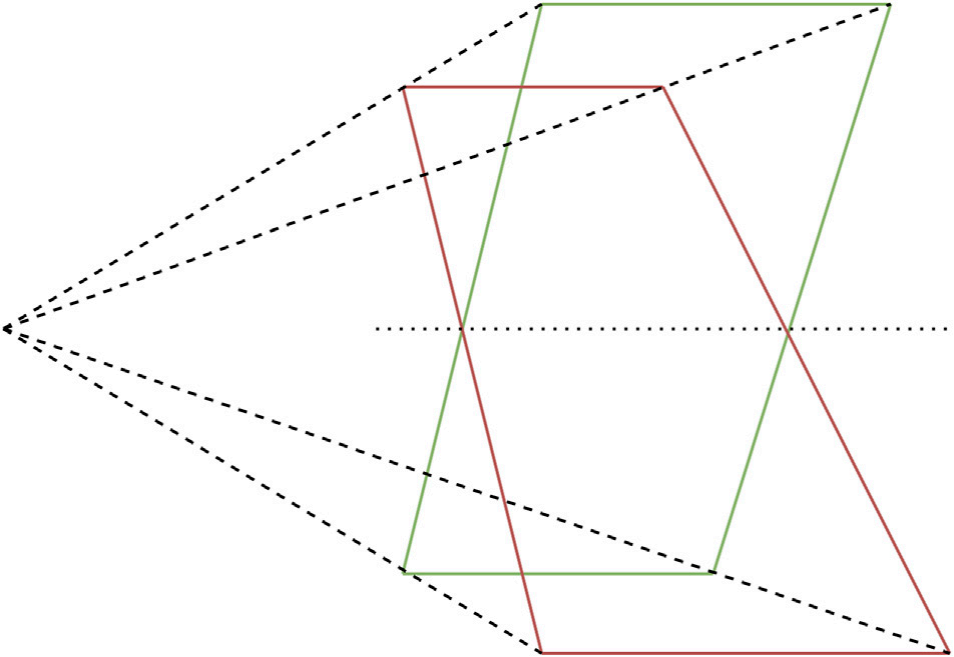
Pose ambiguity in planar feature points.

A geometric representation of pose ambiguity is shown in Figure 1. When only the depicted co-planar features are provided as input to the PnP algorithm, both the pose depicted in green as well as the pose depicted in red is mathematically a valid solution. However, only one of the two poses can be correct in a physical sense. Pose ambiguities exist in many use cases, even including systems utilising cameras with large field of view (FOV) at close range (Schweighofer and Pinz, 2006). If the pose solution is found by minimising some error function, this ambiguity expresses itself in the existence of up to two local minima in the error function. With increasing noise on the system input (e.g., due to increasing distance between camera and target) the mathematical difference between the correct pose and its ambiguous counterpart decreases, thus increasing the severity of the pose ambiguity problem (Jin et al., 2017). Algorithms have been proposed that iteratively find both minima and use an error function to estimate which of the solutions corresponds to the global minimum (Schweighofer and Pinz, 2006; Oberkampf et al., 1996). However, this approach can still cause problems if the absolute values of the minima are closely together due to weak perspective projection effects or noisy measurements.

In addition to pose ambiguity, the pose solution under frontal observations of planar markers is subject to performance loss due to the lack of perspective projective effects (Abawi et al., 2004; Uematsu and Saito, 2007; Kalaitzakis et al., 2021). Since the perspective projections of two distinct but both frontal poses of planar features are similar and measurements are non-ideal (i.e., include positional noise of feature points on the image plane), the found pose solution is likely to be the same. Due to the lack of a distinct signal from the fiducial marker, this cannot be mitigated by algorithmic improvements.

To solve these issues, encoding markers have been proposed. As defined by Bruckstein et al. (2000), these markers directly encode pose information in terms of grayscale, color or temporal signals. This definition is extended here to include markers that encode pose information using a “spatial” signal e.g., a feature that has a variable position on the image plane of the chaser sensor dependant on relative pose. Thus, the definition of an encoding fiducial is: *Fiducial markers whose appearance changes beyond perspective effects with the relative pose of the viewer with respect to the marker.*

Research into encoding fiducials has been limited. In a 1979 patent by Bergkvist. (1979), a device was proposed that provides relative navigation information using Moiré patterns. In a 1984 patent by (Kunkel, 1987), a fiducial that utilises the shadow caused by an illuminating element on the viewer was proposed. A set of reference points on the base of the extended element allowed for pose determination by the viewer by the looking at the extension of the shadow, similar to a sundial. A number of unpowered encoding elements were proposed by Bruckstein et al. (2000). The proposed fiducials include a fiducial with serrated surfaces that appear to change “grayness” based on the viewing angle, a sundial inspired fiducial and a fiducial based on the compound eye of insects such as the Mantis.

A range of different versions of encoding elements utilising lenticular lenses were proposed since 2012 in Tanaka et al. (2012a); Tanaka et al. (2014); Tanaka et al. (2017). These encoding elements utilise an array of lenses on a stripe pattern, producing the effect of a travelling stripe with respect to relative pose of the viewer. The encoding elements solve the problem of pose ambiguity as well as the problem of planar fiducials with respect to the loss of precision in frontal observation. However, these elements only enable retroactive pose correction after the application of a PnP algorithm, increasing the pose estimation algorithmic complexity.

In addition, Tanaka et al. (2012b, 2013) published a fiducial design based on an array of microlenses. This encoding element has a moving cross in two dimensions. Similar to the lenticular lens fiducial, the encoding elements of this fiducial only enable retroactive pose correction.

A coloured pattern in conjunction with lenticular lenses to create a fiducial element that changes colour based on the relative pose of the viewer was proposed (Schillebeeckx et al., 2015; Schillebeeckx and Pless, 2015). A similar design was patented by Larsen (2014). Due to the encoding of the relative pose in colour instead of a moving element the required surface area for the encoding element is relatively small. However, the authors report a slightly lower performance with respect to the Tanaka et al. lenticular lens based marker. In addition, the use of colour as encoding element can be challenging in many illumination conditions. Furthermore, Schillebeeckx and Pless (2016) proposed an encoding element that hashes the relative pose of the viewer using an array of microlenses and a bit pattern underneath the lenses. The authors claim similar performance to existing encoding markers.

While the performance of pose estimation systems using planar fiducial markers can be greatly improved by encoding additional information in the fiducial signal, current encoding fiducials are complex and make use of optical elements such as lenses. To the knowledge of the authors of this paper, no work has been published on an encoding planar fiducial without such optical elements. Furthermore, current encoding fiducials require custom pose estimation software beyond PnP algorithms to correct the found pose solution with the encoded information.

Considering these observations, we present a passive, planar encoding element that requires no optical elements and encodes *virtual points* behind the plane of the fiducial in its signal. We define a virtual point as a point in space for which a fiducial provides features to the viewer, without physically extending to that point. Rather than post-processing the found relative pose with the encoded information, our system is able to directly utilise the encoded additional information in a PnP solver. Furthermore, our encoding element solves both the issue of pose ambiguity and loss of precision under frontal observations. We verify the performance increase our encoding element provides to existing planar fiducials using virtually generated data and qualitatively validate our system using experimentally acquired data. While we test our encoding element in combination with an ArUco marker, our encoding element works in principle with *any* planar fiducial. Therefore, the contribution of the work presented here is not an end-to-end fiducial, but rather an encoding element that aims to improve the performance existing planar fiducials.

## 2 Materials and methods

In this section, we present our encoding element design and end-to-end fiducial marker system design. In addition, we present our software pipeline for pose estimation using our encoding element. We also show the experimental setups of both the virtual and physical experiment used to evaluate the performance of our fiducial marker and qualitatively validate our system.

### 2.1 Novel encoding element

**FIGURE 2:**
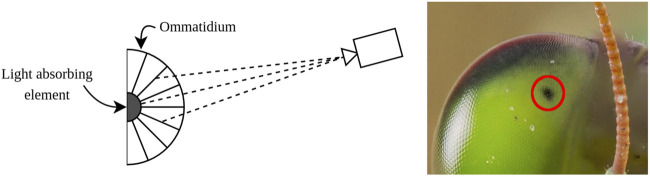
Schematic representation of the compound eye of a Mantis. The black pupil is only visible through the ommatidia that are aligned with the viewer. Based on Bruckstein et al. (2000).

As stated above, Bruckstein et al. (2000) proposed a fiducial based on the compound eye of a praying Mantis. A schematic of the compound eye is shown in Figure 2. A Mantis eye has an apparently moving black spot which follows the viewer, called a pseudopupil. While the pupil moves with relation to the relative pose of the viewer, there is no physical movement of a pupil in the eye of the insect. The apparent movement is caused by the geometry of the eye, which consists of long cylinders called *ommatidia*. Only in the ommatidia that are viewed in line with the line of sight of the viewer, the bottom of the ommatidia can be seen. Since this bottom absorbs light, no light is reflected to the viewer direction causing the black spot (Zeil and Al-Mutairi, 1996).

The idea of using a similar principle is interesting for pose estimation fiducials: the Mantis eye encodes relative pose information in a small form factor. However, the concept as proposed by Bruckstein et al. is complex and non-passive. The authors propose the use of a light source at the centre of the fiducial and optical elements that allow only certain wavelengths of light to pass, making the fiducial change colour when perceived from different relative angles. Apart from complexity, this proposal does not allow for scaleability. In addition, the use of colour as encoding medium is not feasible in many applications.

Thus, a different approach is chosen. Instead of using colour as the encoding medium, it is for many applications more beneficial to use a spatial signal to encode pose information (especially applications with adverse or unpredictable illumination conditions). Realising this, it is also necessary to flatten and elongate the “eye”. Since the information will be encoded in a spatial signal, there needs to be enough resolution on the image plane to do so which causes the need for an elongated marker. Furthermore, the design of the Mantis eye as it appears in nature is *inverted*: the encoding element has a reflective element at the bottom of an ommatidium and absorbing elements around the edges. This produces a reflective pseudopupil that appears on a black background. As an added benefit, it removes the need for any optical or active components on the encoding element. However, an additional requirement is imposed on the viewer: an illumination source parallel to the viewing direction should be applied to ensure proper illumination of the reflective ommatidium bottom.

**FIGURE 3:**
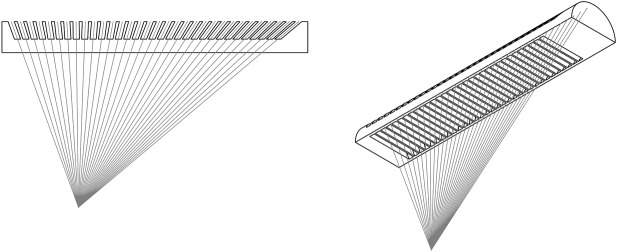
Design drawing showing the virtual point projected by the encoding element. The virtual point is placed off-centre, to increase the FOV of the fiducial marker implementation.

The design of the encoding element is shown in Figure 3. Based on the eye of a praying Mantis, it is a flat and one-dimensional interpretation of the insect eye. The encoding element does not use any optical element such as lenses, is scalable and can be made as a single part. When viewed with appropriate illumination from the viewing direction, a “pseudopupil” or blob encodes pose information by its centroid position on the encoding element.

**FIGURE 4:**
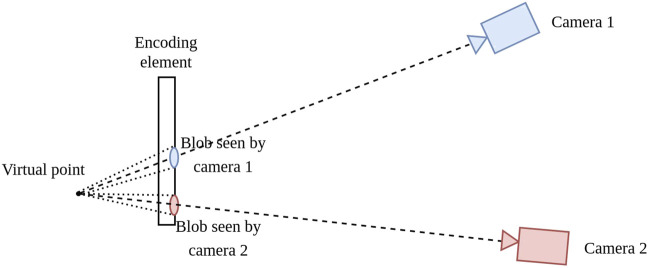
Working principle of the virtual point. The encoding element projects a physical location in space at depth on the image plane without requiring an actual protrusion to that location.

Since the element encodes an actual point in space, the projection of this point on the element’s plane and subsequent projection on the image plane is equivalent to the projection of an actual point at the physical location of the virtual point. This is shown in Figure 4. In other words, our encoding element is *equivalent* to using a protrusion. The virtual point can directly be used as a point in space in the PnP algorithm. This is opposed to current encoding fiducials, which retroactively correct the calculated pose by using an approximation function of the encoding signal with respect to the relative pose.

### 2.2 Encoding element design parameters

The FOV of the encoding element is dependant on several tuneable design parameters. To highlight some of the tradeoffs that are present in choosing the appropriate dimensions of the encoding element, some geometric parameters are developed into equations for the marker FOV here.

**FIGURE 5:**
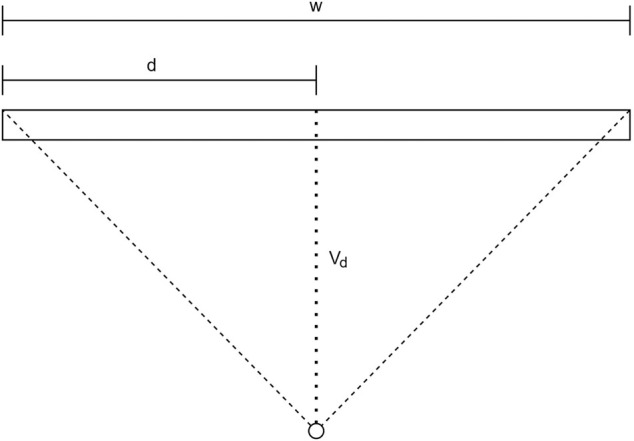
Schematic representation for the FOV calculation of an encoding element.

In general, with a larger distance between the virtual point and the plane of the encoding element (denoted by *V*_*d*_ in Figure 5), the “resolution” of the encoding element increases. In other words, the distance travelled by the blob on the image plane per degree is increased with an increased *V*_*d*_. However, increasing this distance comes at a penalty of a lower FOV of the encoding element.

The FOV can be expressed as a function of the width *w* of the encoding element and the depth of the virtual point *V*_*d*_. This can be expressed as follows:

$$FOV={tan}^{-1}\left(\frac{w}{V_{d}}\right)$$ (1)

Note that in the equation above, a margin should be taken from the FOV in order to account for some distance from the edge of the marker where blob cutoff occurs. In the encoding elements presented here, the virtual point has been moved from the centre of the marker. This has been done to make the FOV asymmetrical, which allows overlap between the FOV of the two parallel encoding elements. This increases the overall FOV of the entire encoding marker system. Now the equation for the FOV of a single encoding element becomes:

$$FOV={tan}^{-1}\left(\frac{d}{V_{d}}\right)+{tan}^{-1}\left(\frac{w-d}{V_{d}}\right)$$ (2)

For the current configuration, *d* = 0.25*w* and *V*_*d*_ = 2*d*. This gives the entire encoding element a FOV of 82.9°. It is estimated that the lateral FOV is around 100° in the current configuration.

Combining these rotations gives an expression of the FOV of an encoding element as a function of pitch and yaw:

$$1=\frac{\psi^{2}}{FOV_{\mathrm{lat}}^{2}}+\frac{\theta^{2}}{FOV_{\mathrm{long}}^{2}}$$ (3)

Where *ψ* is the yaw defined in the lateral direction of the encoding element and *θ* is the pitch defined in the longitudinal direction of the encoding element. In this case. *FOV*_*long*_ is the longitudinal FOV of the particular side of the encoding element where the FOV is calculated.

### 2.3 End-to-end encoding fiducial marker

**FIGURE 6:**
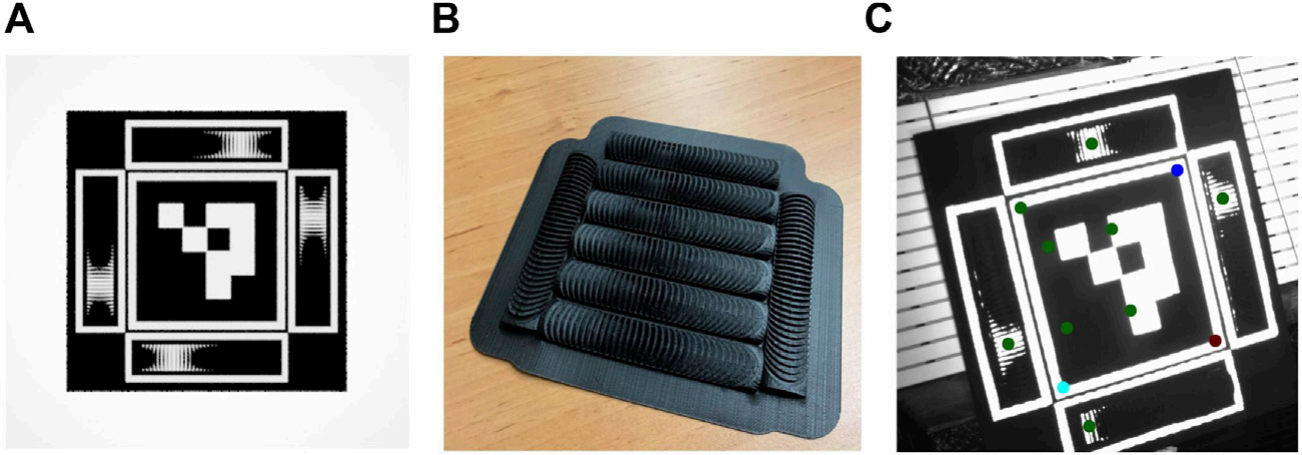
End-to-end implementation of the proposed encoding element in a fiducial marker. **(A)** Render of ArUco with additional proposed encoding elements. **(B)** 3D printed encoding elements **(C)** Output of feature extraction. Note that the bottom blob fails our verification step due to close proximity to the edge of the marker, thus both virtual points on the left are not passed to the pose estimation block.

Since additional feature points are required to provide a 6 degree of freedom pose estimate, the encoding element requires additional features. Furthermore, a fiducial for monocular pose estimation should be uniquely identifiable. To this end, existing fiducials use bit patterns to encode an unique identity for each marker. For our current implementation, we add an ArUco marker (Garrido-Jurado et al., 2014) to provide the bit pattern and the additional feature points required, as shown in Figure 6A. The ArUco marker was chosen since it is a representative and widely used marker. It should be noted that our marker design is not limited to the ArUco, any bit pattern with at least 4 co-planar feature points could be applied. Our end-to-end fiducial prototype is appropriately named the Mantis Marker.

Each Mantis Marker consists of four encoding elements in addition to the ArUco marker. Two encoding elements make up a virtual point in three-dimensional space. By placing the virtual points of the encoding markers off-centre (as shown in Figure 3) and using 4 markers, the overall FOV of the marker is increased due to overlapping angular reach. With this design, a total of four virtual points (equal to the number of possible combinations of the two horizontal and vertical elements) are added to the four features of the ArUco marker.

The encoding elements of the Mantis Marker prototype were produced using a consumer grade 3D printer. A set of elements produced using such a printer is shown in Figure 6B. The dimensions of the prototype are driven by the resolution of the 3D printer. Alternatively, the simple geometry of the encoding element allows for other production techniques such as milling which could allow smaller dimensions.

### 2.4 Software

A fiducial marker system requires a feature extraction function and a pose estimation function to convert the input of the system (the fiducial image) to the output (the relative pose). The algorithmic steps of the fiducial marker system can be summarised as follows:

1. Find square fiducial and utilise bit pattern to identify fiducial
2. Extract co-planar features (corners)
3. Utilise relative location of corners and perspective projection to generate mask for each encoding element
4. Attempt blob detection for each encoding elements
5. Verify found blobs inertia, area and relative location to element edge
6. Calculate virtual point coordinates
7. Pass found feature locations (co-planar corners and virtual point) in image frame and world frame to PnP solver
8. Find relative pose

Since our prototype utilises the ArUco marker, its features are readily extracted from the input image. The ArUco corners for both the standalone marker as well as the Mantis Marker are refined using the Apriltag 2 approach detailed in Wang and Olson (2016). This increases the performance of the planar fiducial as well as the Mantis Marker and is therefore a more accurate representation of the state of the art pose estimation performance. Once the ArUco features are extracted by the fiducial’s feature extraction software, the blobs produced by the encoding elements need to be extracted. These blobs encode the location of the virtual point on the image plane. Using the relative location and perspective projection of the ArUco marker with respect to the encoding elements, a mask is generated for each encoding element starting with the top element and moving clockwise. For each encoding element, a Gaussian blur is applied to the mask to smooth out noise and a blob detection algorithm is applied. The detected blobs are verified to comply with the expected area and inertia. Next, the blob centroids are verified to be located far enough from the edge of the encoding element, to prevent blob cutoff and subsequent loss of precision (LeCroy et al., 2007). If a blob fails verification, a zero is passed to the pose estimation algorithm. If a blob is verified successfully, the horizontal and vertical coordinates of the blob on the image plane are passed.

The location of the blobs on the image plane is found directly from the blob detection algorithm. However, since each encoding element encodes one-dimensional information (each blob can only move in a straight line), the coordinates of a blob on the image plane are only sufficient to encode a single coordinate of a virtual point. Hence, the blob coordinates from two perpendicular encoding elements encode a virtual point.

In order to calculate the coordinates of a virtual point on the image plane, first the slope of the ArUco sides is calculated. Next, the following equations are applied for the virtual points for which the respective blobs were found:

$$v_{i}=a_{v}*u_{i}+b_{j}$$ (4)

$$v_{j}=a_{h}*u_{j}+b_{j}$$ (5)

$$b_{i}=v_{i}-\left(a_{v}*u_{i}\right)$$ (6)

$$b_{j}=v_{j}-\left(a_{h}*u_{j}\right)$$ (7)

$$u_{k}^{v}=\frac{b_{i}-b_{j}}{a_{h}-a_{v}}$$ (8)

$$v_{k}^{v}=a_{v}*u_{k}^{v}+b_{i}$$ (9)

where i=1,3 and j=2,4 and k=1,2,3,4 (10)

Where *u*_*n*_ and *v*_*n*_ are the horizontal and vertical coordinates of the *n*th blob in the image frame in pixels, *a*_*v*_ and *a*_*h*_ are calculated vertical and horizontal slopes of the ArUco, *b*_*n*_ is the *y* axis intercept of the line parallel to the relevant ArUco slope and crossing the blob centroid and $u_{k}^{v}$ and $v_{k}^{v}$ are the horizontal and vertical coordinates of the *k*th virtual point. The output of this calculation are the virtual point coordinates on the image plane. The output of the feature extraction software is shown in Figure 6C.

The pose estimation software takes as input the found points in the image frame (both virtual points and fiducial corner points), the dimensions of the ArUco as well as the location of the virtual points in the world frame (which are a function of the encoding element dimensions). Finally, the camera intrinsics of the calibrated camera are passed as input. The intrinsics can be found in a calibration procedure using an approach proposed by Zhang (2000). This approach can be summarised as taking a range of images at different relative poses using a “chessboard” i.e., a plane with a number of alternating black and white squares. Using an initial guess of the intrinsic parameters found by finding the homography between the chessboard and its image, the intrinsics are found by iterating until the reprojection error reaches a certain threshold.

Any non-planar PnP solver can be used in combination with the proposed fiducial. For the experiments performed, a solver is used which initialises a pose solution using homography and a subsequently optimises the pose using a Levenberg-Marquardt optimisation.

### 2.5 Performance benchmarking and system validation

To benchmark the performance of the Mantis Marker and its software, a dataset was generated using 3D modelling software. Where historical encoding fiducials are difficult to model due to the use of optical elements, the ommatidia of the Mantis Marker can be modelled accurately. In addition, a physical experiment was performed. The goal of the performed physical experiment is to validate the marker system in a real-life scenario and to validate the created virtual dataset.

#### 2.5.1 Virtual experimental setup

**FIGURE 7:**
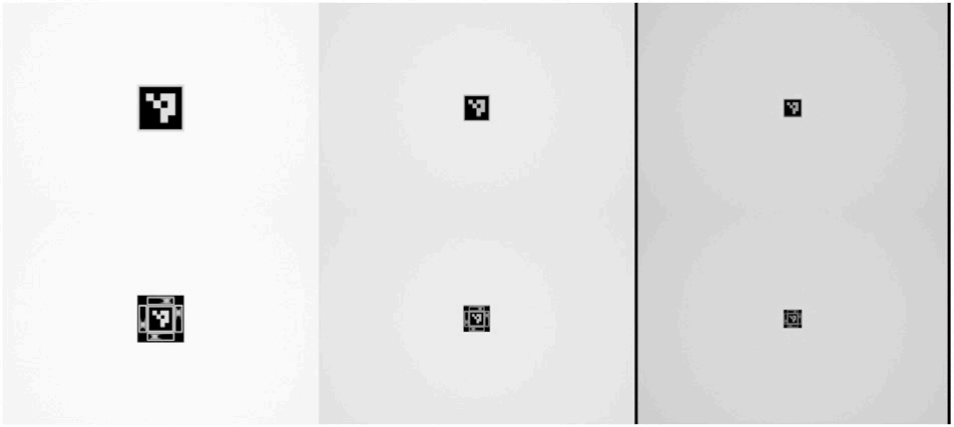
Sample of test images at ranges of 1.3, 2.3 and 3.3 m.

The Mantis Marker with ArUco implementation and an ArUco fiducial with identical dimensions (2.5 × 2.5 cm) were tested at a distance of 1.3, 2.3 and 3.3 m. The virtual points were located at a distance of 6.25 mm from the fiducial plane. A sample of the generated images is shown in Figure 7. For each distance and for both markers, 900 images were generated at a rotational range around the pitch axis, from −45 to 45°, stepwise incremented by 0.1° per image. i.e., we generated a total of 5,400 virtual images for our experiments.

For the virtual experiments, a Blender model was used. Blender is a free and open-source “3D creation software suite” (The Blender software can be found at: https://www.blender.org/). It allows for the rendering of images using ray tracing, thus enabling the testing of fiducials to a degree of realism.

The virtual dataset is generated using a detector with a resolution of 720 × 480 pixels. The camera has an FOV of 7.4° horizontal and the simulated detector dimensions are 4.512 × 4.988 mm. For each dataset generation, the CAD design of the encoding element was loaded into a mock-up of the complete fiducial (including an ArUco marker). Due to the required viewer-centred illumination, a virtual spotlight was programmed to coincide with the camera principal point and imaging direction.

Blender is scriptable in the Python programming language, and a script was implemented that automatically takes a range of images for set fiducial orientations and relative positions. For each image in the test dataset, the ground truth for the relative pose was saved.

#### 2.5.2 Validation experimental setup

The Mantis Marker was additionally tested in a relative navigation scenario for spacecraft at the Orbital Robotics and GNC Lab (ORGL) at ESA ESTEC. Zwick et al. (2018) extensively describe the capabilities of the lab. A ceiling mounted robotic arm was used to precisely control the relative movement of the camera with respect to the Mantis Marker. This experiment was intended for the qualitative validation of the system in a challenging relative navigation environment.

**FIGURE 8:**
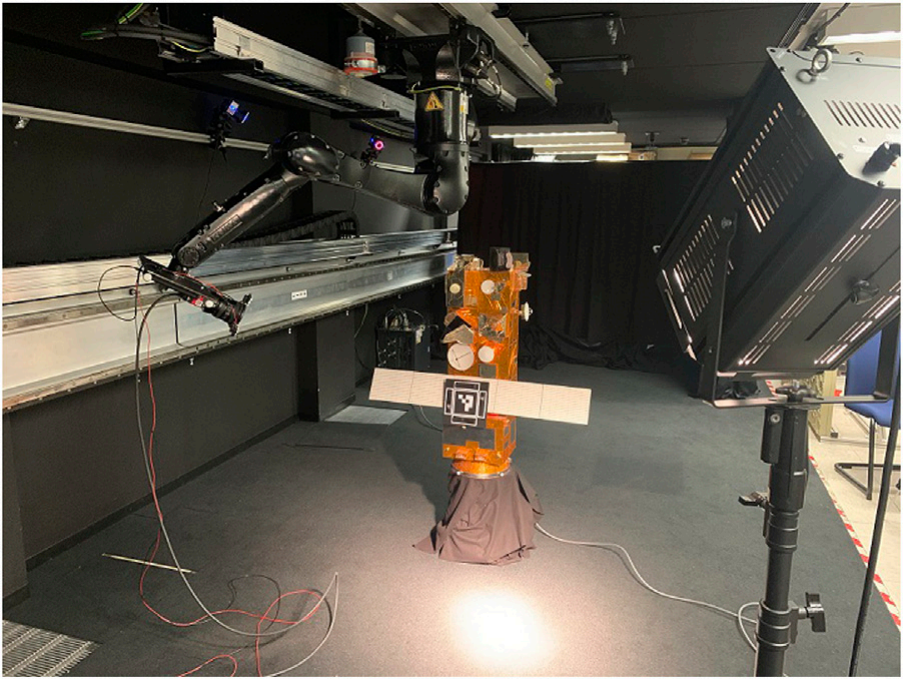
Test setup for the validation experiment. To the left, the robotic arm with the attached camera and illumination system is shown. In the centre, the mockup of a spacecraft with the applied Mantis Marker is visible. On the right the spotlight is visible. Note that during testing the illumination environment is controlled, not shown here.

The test setup (shown in Figure 8) consists of a camera attached to the end effector of the ceiling-mounted robotic arm, configured in an open-loop system integration. To this camera is an illumination system attached which illuminates the target. A 20 × 20 cm prototype of the Mantis Marker was manufactured using 3D printing. A mock-up of Envisat was used to mimic typical shapes and reflections present around a fiducial target on a spacecraft. All results were recorded using Simulink.

A camera trajectory was used that consisted of three 180° passes of the fiducial marker at different heights to simulate a range of combined pitch and yaw rotations. Each pass is performed two-way, in total six one-way passes are made for a single run.

The detector that was used for the experiments is the Prosilica GT4096 NIR from Allied Vision. (2021). This is a monochrome detector that is able to capture a spectrum of light including near-infrared. Its key specifications are listed in Table 1. Attached to this detector is the Canon EF 24–70 mm f/4L IS USM lens. This is a zoom lens with variable focal length and adaptable focus. For the used focal length of *f* = 24 mm, the FOV is 42.01 deg.

**TABLE 1:** Prosilica GT4096 NIR specifications [Allied Vision (2021)].

Parameter	Value	Unit
Resolution	4,096 × 4,096	Pixels
Sensor type	CMOS	—
Sensor size	Type APS-H	—
Pixel size	4.5 × 4.5	*μm*
Temporal dark noise	28.2	electrons
Max. frame rate at full resolution	7.18	fps

**FIGURE 9:**
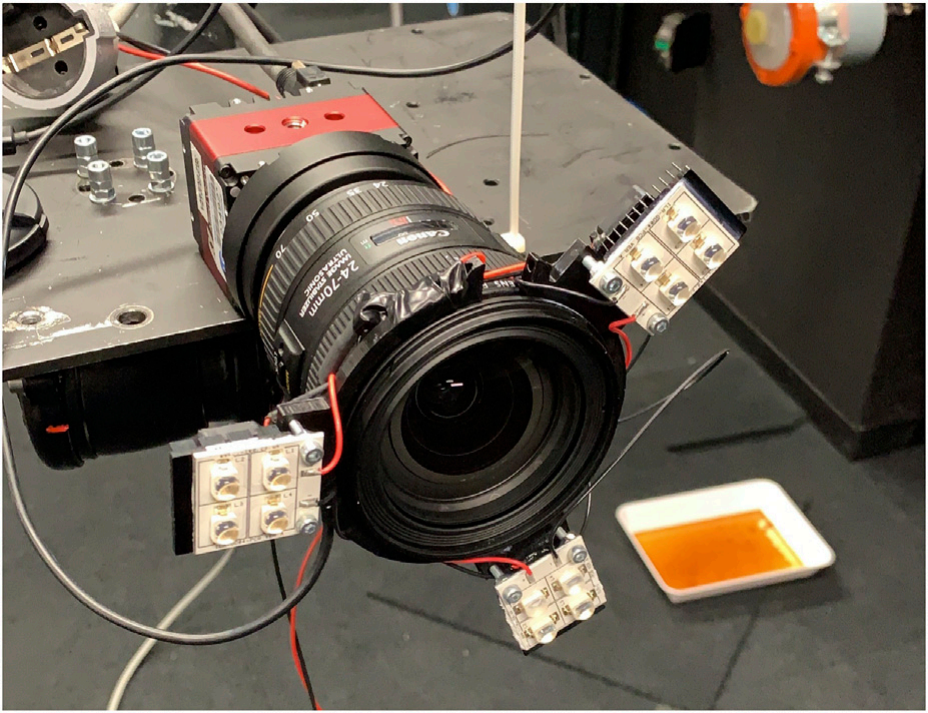
LED mount as applied to the camera.

A custom illumination system was made to illuminate the target (shown in Figure 9). This illumination system consists of 3 clusters of LEDs with a half angle of 12°. The LEDs are powered by a current limited power supply and mounted using a custom-designed and 3D printed camera mount. To this mount, active cooling is applied using fans.

Since the camera records data at very high resolution and the distance between camera and marker is small, the internal planar fiducial causes no failure of the pose solution for the taken data. In order to provide a comparison of data to the virtual dataset, the image needs to be scaled down to be representative of the virtual data.

**FIGURE 10:**
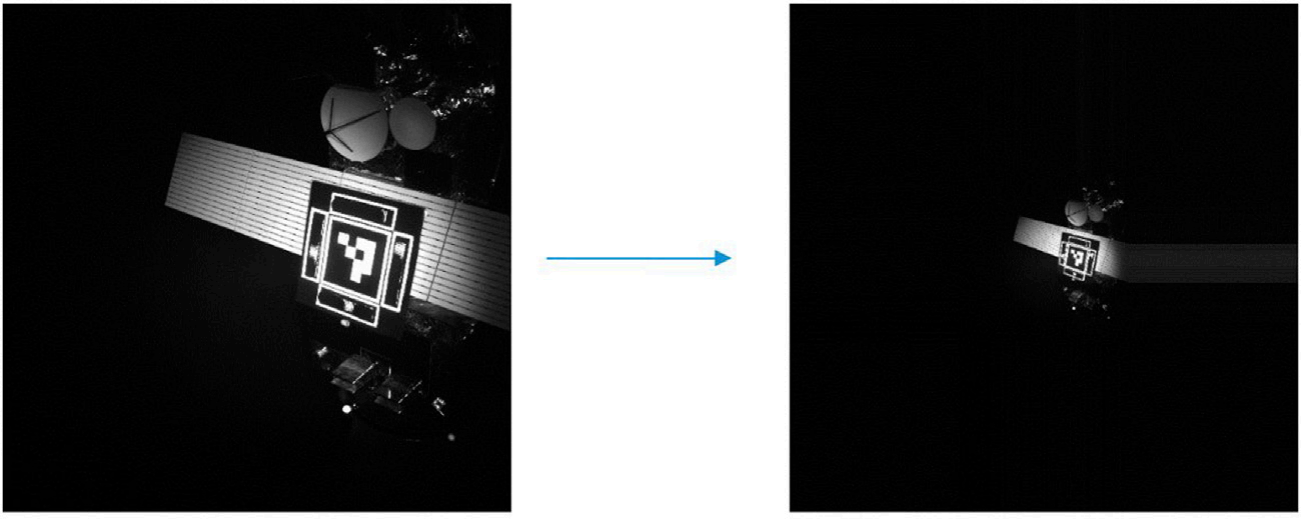
Image scaling to enable Blender model validation.

To do so the image is scaled down and padding is added by repeating the outer pixel values to have the fiducial be of comparable size relative to the images generated by the Blender model. Next, the resolution is strongly reduced to match the resolution of the virtual dataset. Finally, the camera intrinsics are scaled according to the new image resolution and virtual pixel size. An example of the generated data is shown in Figure 10.

## 3 Results

**FIGURE 11:**
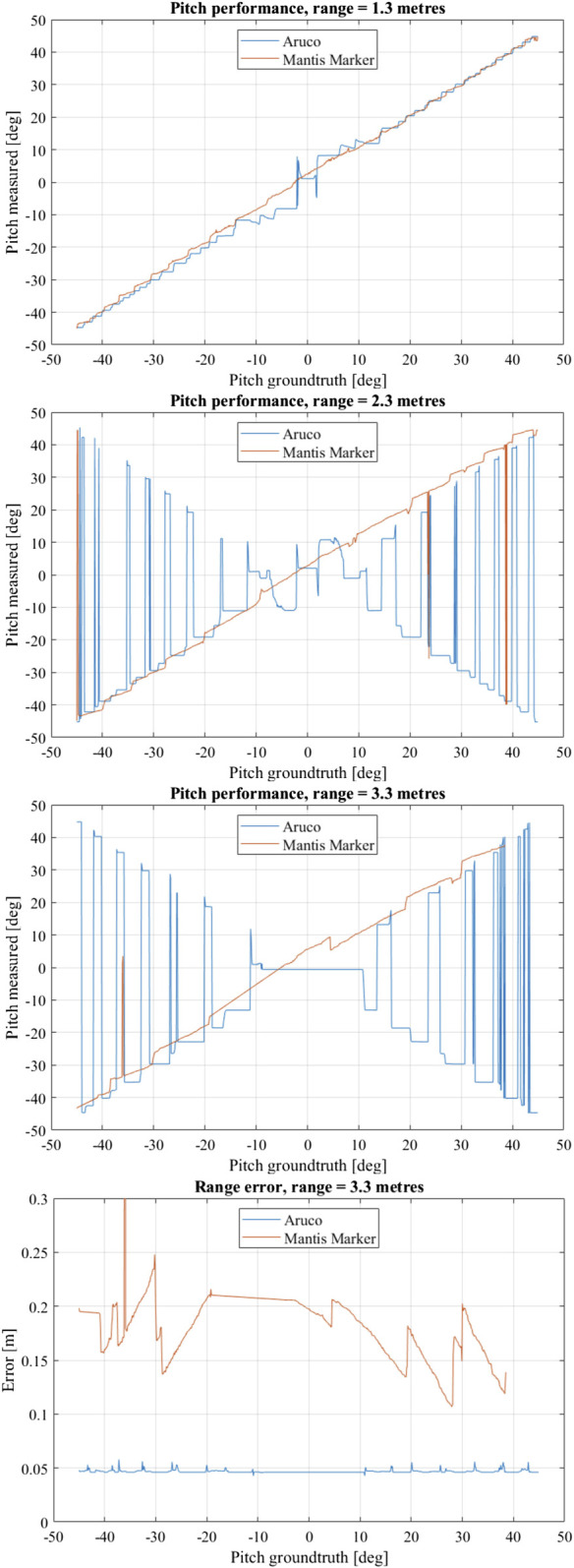
Performance evaluation results of virtual experiment. Shown are the pitch measurement vs. pitch ground truth at 3.3, 2.3 and 1.3 m, respectively. For these measurements, the fiducial was only rotated along the pitch axis. In the bottom graph, range error vs. pitch ground truth at 3.3 m is shown.

The experimental results for the virtual experiments are shown in Figure 11. At a range of 3.3 m the Mantis Marker greatly outperforms the ArUco marker in terms of pose stability as well as accuracy under frontal observations. The ArUco marker system is unable to distinguish any pose between −10 and 10° pitch due to lacking perspective effects. Furthermore, the ArUco marker system suffers from many pitch solution flips due to pose ambiguity. This behaviour is not observed in the Mantis Marker.

A lack of pose solutions for the Mantis Marker for measurements above a positive yaw of 38° is observed. Notably, the ArUco solutions for this pitch range also oscillate severely. It appears that the ArUco marker used in this particular setup has a asymmetry in the detectability of the encoding bit pattern. At the very limit of the experimental domain in terms of range and attitude the reduced size of the internal ArUco of the Mantis Marker with respect to the benchmark ArUco, causes the feature extraction software to be unable to find the marker.

At 2.3 m, the ArUco marker also suffers from pose ambiguity as well as reduced precision in frontal observations and no significant change is measured with respect to the 3.3 m range. The Mantis Marker has a small number of pose jumps, where the error is equivalent to the error of the ArUco marker in terms of pitch while the range error is large. In these rare cases, the pipeline is unable to find any blobs.

The ArUco solution at 1.3 m is much improved in stability with respect to the measurement distance of 2.3 m. However, the Mantis Marker still shows more stability for pitch measurements. Especially under frontal observations, the ArUco relative pose solution remains with inaccurate.

Notably, the range estimation error of the ArUco marker with respect to the Mantis Marker is significantly lower and more stable, also shown in Figure 11. This is due to the design of our Mantis Marker prototype: the internal ArUco marker in our design provides smaller planar features with respect to the reference ArUco marker due to its reduced size. This can be solved by a design improvement, namely using the corners of the complete marker as feature points in the PnP, therefore effectively increasing the Mantis Marker feature points to the same dimensions as those of the ArUco. This would limit the amount of available bit patterns for marker identification, which is not an issue for applications where an encoding element is required to improve pose estimation performance. In these applications, the number of available markers is inherently limited. As an added benefit of this design improvement, the relative pose estimation performance of the Mantis Marker with respect to the benchmark ArUco would increase.

**FIGURE 12:**
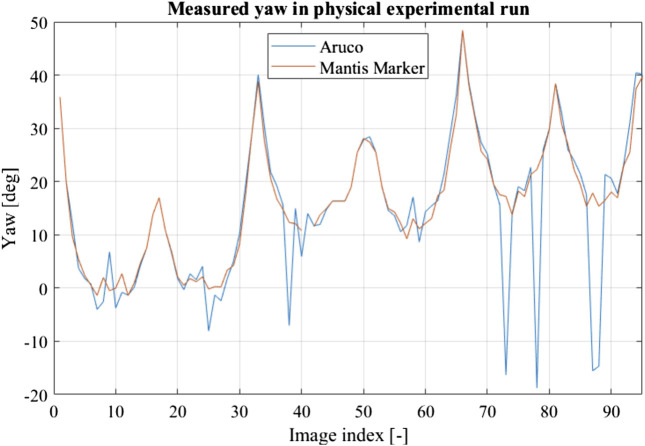
Result of validation experimental run using a robotic arm. The ArUco marker without encoding element shows both loss of precision in frontal observations and pose ambiguity, similar to the created virtual dataset.

The internal ArUco marker performance was compared to the performance of the same marker including the virtual points provided by the encoding elements in the validation experiment. This result is shown in Figure 12. The ArUco without virtual points shows a loss of precision under frontal observations as well as pose ambiguities while the pose solution found using the virtual points is stable. This result validates the virtual model, as well as the application of the Mantis Marker in a real-world scenario.

**FIGURE 13:**
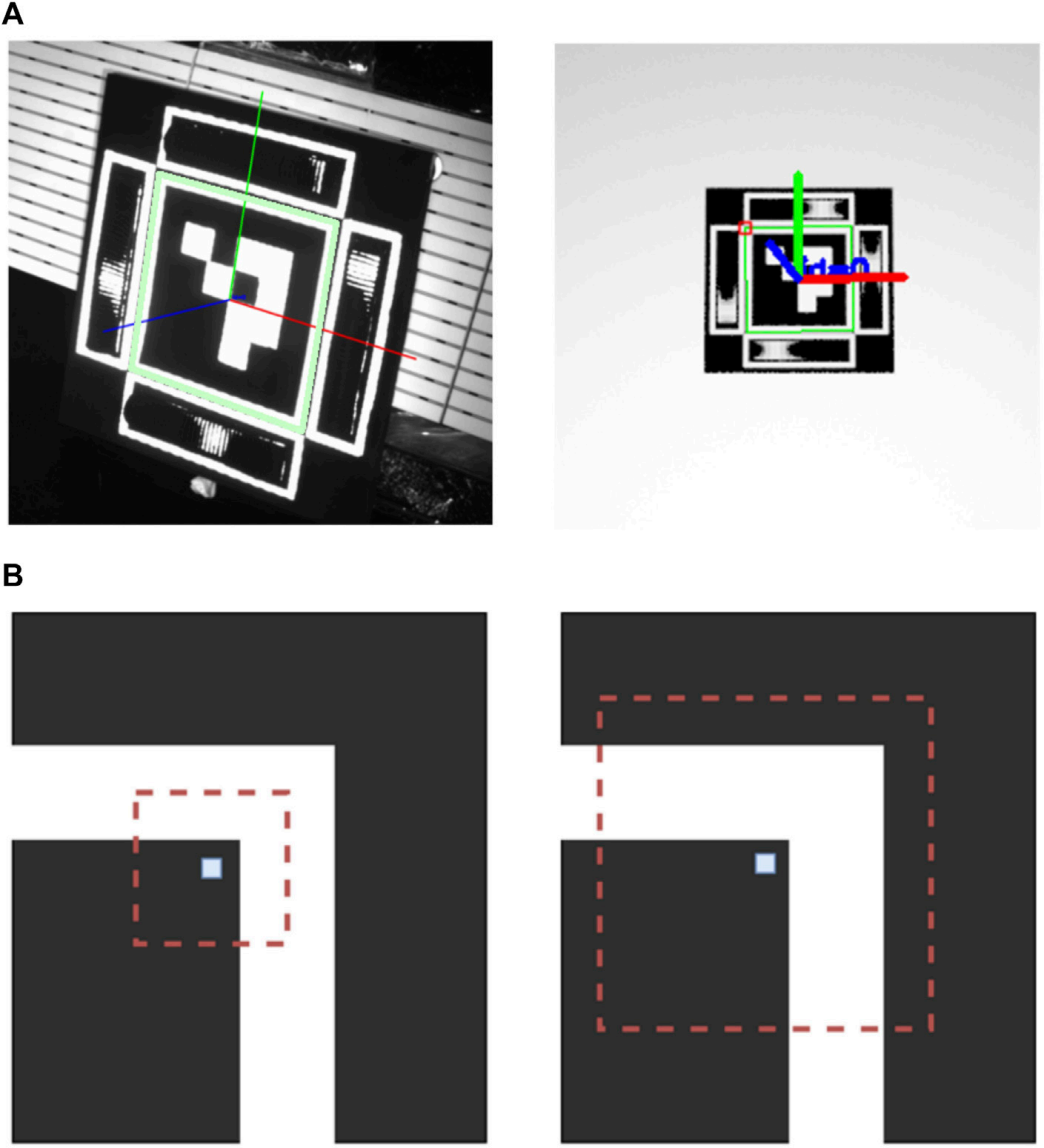
**(A)** Faulty corner detection of ArUco marker in validation and virtual experimental data. A green border can be seen around the outside edges of the white border around the ArUco marker. This border should be present at the inner edges. **(B)** Corner ambiguity in the corner detection of the ArUco marker. The central pixel is the first estimation of the detected corner, with the red window being the search area. In the left image, no ambiguity is present since only one corner is in the search window. In the right image, the search window includes two corners and is thus ambiguous. Illustration based on Deom et al. (2019).

In both the virtual performance measurements as well as the validation experiments, a failure mode of the Mantis Marker as observed caused by the wrongful detection of the ArUco corners. This failure mode was observed to be most prevalent at close range and frontal observations. In Figure 13A, the failure mode is shown. The green border around the ArUco marker should be located around the inner corner of the marker, not at the outside corners. The cause for this failure mode was identified to be an ambiguous corner refinement.

Ambiguous corner refinement occurs when the feature extraction method comes across an ambiguous corner detection. This can be caused by two distinct factors: illumination conditions and marker design. In our experimental setup, faulty corner detection was most prevalent in frontal observation where strong illumination causes the correct corner candidates to be less pronounced. Secondly, in the tested prototype a small gap was present between the border around the ArUco marker and the border around the Mantis Marker. At close range, this can cause ambiguous corner detection due to the search window of the corner detection algorithm including two corners, as depicted in Figure 13B. This effect was also described in Deom et al. (2019). Normally, this becomes more prevalent at larger range where the search window is proportionally larger than the edges of the marker. However, in the case of the used test setup, the gap was too small to be distinguished by the feature extraction pipeline at the medium and long range. Ambiguous corner detection has a consequence of a major loss of accuracy of the pose estimation. Both range and orientation can become distorted and the failure mode causes the Mantis Marker to be undetectable in nearly all cases since the relative location is faulty. The mitigation of this failure mode is straightforward: if an ArUco is used as primary marker in future work, this marker should include a wider border around its edges. In addition, the white border around the encoding elements can be removed if only the relative location of the encoding element with respect to the ArUco corners is used in the feature extraction pipeline.

**FIGURE 14:**
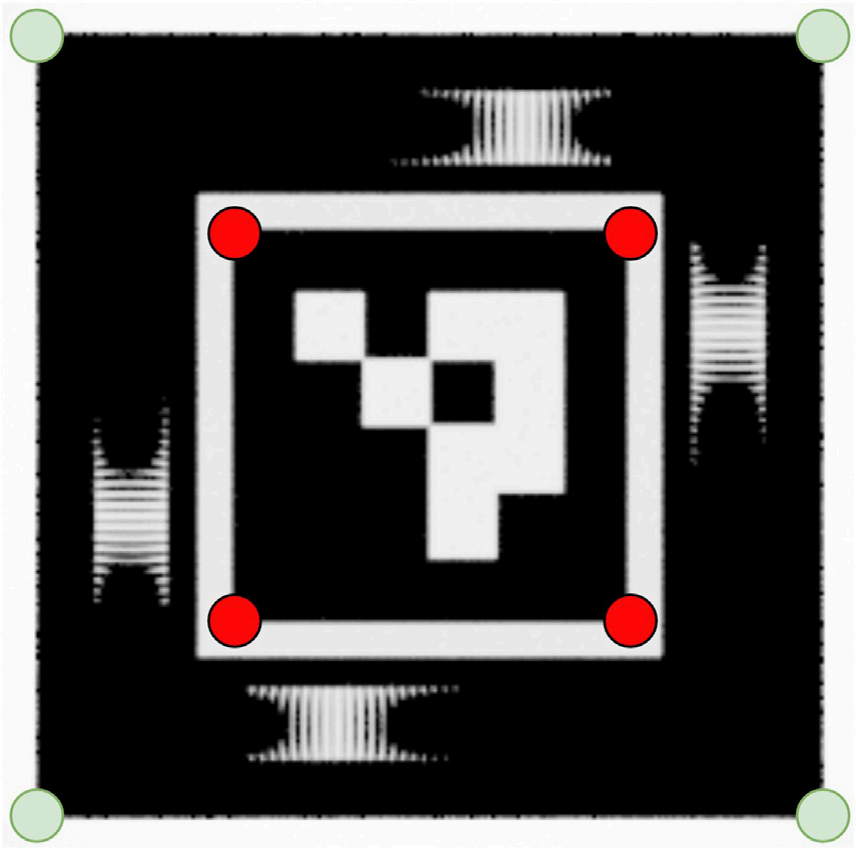
Proposed design improvements for implementation of our encoding element. The white borders of the encoding elements are removed. In addition, the corners of the end-to-end fiducial should be used as features for input to the PnP (indicated by the green markers), instead of the internal ArUco corners (indicated by the red markers) as is the case in the current implementation. This will increase the range performance of a marker that utilises our encoding elements.

The deficiencies in the end-to-end prototype implementation of our encoding element presented here aim to inform the reader on implementation considerations for our encoding element. When adding our encoding element to existing planar fiducials, the design improvements proposed here should be taken into account. We have rendered the proposed design improvements in Figure 14.

## 4 Discussion

With the combination of these results, it can be concluded that the proposed encoding element greatly improves the attitude estimation of planar fiducials. The element prevents pose ambiguity as well as precision loss under frontal observations. Furthermore, the end-to-end implementation of our encoding element with an ArUco marker outperforms a standard ArUco marker in attitude estimation. At larger ranges, the relative performance increase of the Mantis Marker with respect to the ArUco is larger due to the increased likelihood of pose ambiguity.

The contribution of this work is not the Mantis Marker itself, but the demonstration of encoding virtual points in a fiducial signal. By keeping the relative marker dimensions between the planar ArUco and the ArUco with encoding elements constant and comparing the relative performance, the principle and benefits of virtual point encoding were proven. We claim that these encoding elements could be used with *any* currently existing planar fiducial, thus providing a low-cost and simple method for increasing the pose estimation performance of systems that use these markers.

Since the magnitude of the increase in performance is dependant on many factors (such as fiducial size, viewing range, illumination conditions, camera parameters, etc.) the investigation of the performance increase was limited to the ArUco marker in this paper. We believe that this investigation should be performed on a system by system basis, taking into account the specific application environment of a particular end-to-end system. Such an investigation would not only concern the fiducial itself, but also e.g., the camera and illumination conditions.

In application areas where pose ambiguity or lack of precision in frontal observation is likely to occur due to a lack of perspective effects, the addition of our encoding element to an existing planar fiducial provides a significant performance increase. The application area is systems with e.g., low FOV, small marker size, low resolution detector, large range, limited available surface area etc. The encoding of virtual points in a fiducial signal is not a one-size-fits-all solution but will provide significant benefit to a range of fiducial-based relative navigation systems that are applied in constrained environments.

The end-to-end fiducial in the presented configuration has an applicable angle of view of around 45° under rotation around a single axis (pitch or yaw). Under combined rotation of pitch and yaw, this angle of view is reduced to around 32°. Depending on the application, this can be adapted by changing the depth of the virtual point with respect to the fiducial plane. Furthermore, under increased rotations the perspective effects of the ArUco are increased reducing the necessity for an encoding element.

Two design considerations that should be taken into account when including the proposed encoding element in an end-to-end fiducial system were identified. Firstly, the corner features of the end-to-end fiducial should be used in the PnP solution instead of the embedded ArUco corners. This increases the range performance of the fiducial. Furthermore, the white border around the inner ArUco marker should be increased to prevent faulty corner detection. A custom bit pattern and planar feature extraction may solve these issues by removing the ArUco from the end-to-end implementation. In addition, while the encoding element is 3D printable, on consumer printers this yields relatively large encoding elements due to limited printer resolution. In future work, additional manufacturing methods should be investigated to reduce the fiducial size. Alternatively, the number of ommatidia may be reduced to shrink the printable encoding element size.

To conclude, the encoding element presented in this work encodes additional pose information by a moving blob that represents a projection on the fiducial plane of a virtual point behind the fiducial plane. To the best knowledge of the authors, our encoding element is the only encoding fiducial from which the signal can directly be used in a classical PnP solving algorithm. Furthermore, the proposed encoding element is scalable and can be adapted to be used with any planar fiducial. The element provides a low-cost, scalable solution for systems requiring planar but high performance fiducial markers in constrained environments.

## Data Availability

The raw data supporting the conclusion of this article will be made available by the authors, without undue reservation.
